# Ex ante assessment of sustainable marketing investments

**DOI:** 10.1007/s43039-022-00052-1

**Published:** 2022-04-04

**Authors:** Agostino Vollero, Alfonso Siano, Alessandra Bertolini

**Affiliations:** 1 Department of Political and Communication Sciences (POLICOM), University of Salerno, Fisciano, Italy; 2 Department of Economics and Statitistics, University of Salerno, Fisciano, Italy

**Keywords:** Sustainable marketing investments, Ex-ante assessment, Risk/reward ratio

## Abstract

The sustainability revolution, and the positive effects of sustainable marketing on business performance, should lead companies and research to focus more on issues related to assessment of sustainable marketing investments. This conceptual paper is an attempt to provide a contribution on this interdisciplinary topic not yet considered in the literature. We focus on how companies can assess ex ante this type of investment. We propose an adaptation of the risk-return ratio as a tool for ex ante assessment of sustainable marketing investments. We also provide considerations about the risks and rewards of Starbucks’ “Greneer Stores” project. We conclude the study by discussing the implications for managers of sustainable companies.

## Introduction

The sustainability revolution (Edwards, 2005) in recent years has led to more corporate investments in sustainable assets (Pástor, Stambaugh & Taylor, 2021; Lauesen, 2019), in CSR policies and projects, and in sustainable marketing tools (Sims, 1991; Graafland & Smid, 2019). Despite the growing interest among researchers in sustainable investments and the evaluation of their financial performance (Lauesen, 2019; Ameer & Othman, 2012; Busch, Bauer & Orlitzky, 2016; Siano, Vollero, Conte & Sardanelli, 2021), there are no contributions that address sustainable marketing investments. Researchers in sustainable marketing have mainly focused on the contribution of sustainable marketing to company economic performance (Peattie, 2001; Rao & Bharadwaj, 2008; Hunt, 2011), corporate reputation (Fombrun & van Riel, 2004; van den Bosch, de Jong & Elving, 2005), and consumer consumption choices (Rokka & Uusitalo, 2008; Rettie, Burchell & Riley, 2012). There are no contributions in the sustainable marketing literature (Kumar, Rahman & Kazmi, 2013; McDonagh & Prothero, 2014; Kemper & Ballantine, 2019) which explain, define and assess sustainable marketing investments, particularly pertaining to risk assessments (Lintner, 1965; Riggs, 1974). The absence of literature on sustainable marketing investments may be due to its recent emergence, as well as its interdisciplinary nature, which requires the integration of corporate finance considerations with that of marketing (Rao & Bharadwaj, 2008), and, in particular, sustainable marketing.

In this paper, we attempt to contribute to the literature on sustainable marketing by examining investment in sustainable marketing and its ex-ante assessment from an interdisciplinary perspective. Through the design of the theory synthesis proposed by Jaakkola (2020), our paper integrate, in fact, considerations derived from corporate finance into the topic of sustainable marketing.

Because there is no literature on sustainable marketing investments, it is essential to first determine what sustainable marketing investments should mean in order to make the study worthwhile. After this specification, we seek to determine how to assess these investments ex-ante, so that useful indicators can be derived for decision making; and to explain how companies can benefit from ex-ante assessment of these investments, and what considerations should be made by these companies.

Ex-ante assessment of investments, in corporate finance, refers to the prediction of risks and returns on investments (Hirshleifer, 1993; Errington, 1994). The “anticipatory” investment assessment, in fact, outlines the expected losses associated with the expected risks and the expected returns related to corporate investments (Hirshleifer, 1993; Forstmoser & Herger, 2006). For this type of assessment, the risk-return ratio is the appropriate tool (Errington, 1994; Weyns, Perez, Hurewitz & Jenkins, 2011).

Our proposal is to adapt this ratio to the ex-ante assessment of sustainable marketing investments, since it is possible to consider such investments as corporate investments.

The interdisciplinary approach adopted in this research constitutes the most original contribution of the paper. Moreover, we cover a topic that has never been addressed in the literature. It can contribute to creating a research space about this growing phenomenon.

The paper reminder is as follows. Within Sect. 2, we examine the corporate finance literature on investment assessment, the marketing investment assessment, and the sustainable marketing literature. This is done to establish a theoretical foundation and support the subsequent theoretical arguments developed in the paper. In Sect. 3, we will define the research method, clarify the gap in the literature, and formulate consequential research questions. As part of Sect. 4, we provide the results of our research, defining sustainable marketing investment and adapting the risk/reward ratio to the ex-ante assessment of such investments. In addition, considering the “Starbucks Greener Stores” investment in 2018, we will discuss some considerations that the company could have made, in relation to the expected risks and expected rewards, if it had wanted to apply the RRr. In Sect. 5, we report and discuss the implications of the work. The paper ends with a concluding section reporting the limitations and future prospects of the research (Sect. 6).

## Conceptual background

Due to the aforementioned lack of a specific reference literature dealing with sustainable marketing investments and their ex-ante assessment, as well as the inter-disciplinary nature of the topic, in this section we construct a conceptual background that enables us to consider the two theoretical fields of research into which this topic falls: the literature on investment assessment (corporate finance) and the literature on marketing and, in particular, sustainable marketing.

### Assessment of investments in corporate finance

The assessment of investments is one of the most critical aspects of corporate finance (Ross et al., 1999; Vishwanath, 2007). An investment can be defined as the allocation of resources to achieve immediate or later gain, either in more resources or in avoiding risk (Eades, 2018).

The main financial and economic assessment methods for investments are: return on investment, payback period, discounted cash flow, present value and terminal value. Their applicability lies in the prediction of certain dimensions such as outflows (the amount and rate of expenditure), inflows (the amount and rate of income), costs of capital and reinvestment rates (Ross et al., 2005; Vishwanath, 2007). Apart from the purely objective and quantitative parameters and methods related to the financial performance of the investment, an investment choice is usually evaluated in relation to the elements of risk and the expectation of the income effects that will be produced on the results of the organization as a whole (Hussain, 2000). These predictions are based on the uncertainty of competitive environment (Shimpi, 2002; Vishwanath, 2007).

Before the introduction of the Capital Asset Pricing Model (Sharpe, 1964), the dominant paradigm for estimating expected returns places the “cost of capital” of an asset, at the center of investment considerations, which depends primarily on how the asset is financed (Bierman & Smidt, 1966). Gordon and Shapiro’s (1956) model, for example, designates a method for estimating the cost of capital based on the financial structure, in other words, the composition of the sources a company uses to conduct its business (Galbiati, 1999). As Modigliani and Miller (1958) show, in modern finance, the value of a company or asset depends not only on how it is financed, but it is necessary to consider the risk adjustment dimension, assessing how it varies in response to changes in other variables. In fact, every investment project today is evaluated not only in terms of expected return, that is, considering the actual gain from the operation of allocating monetary resources; but also, in terms of risk (Riggs, 1974; Errington, 1994). Risk is a key element to consider in contexts of uncertainty, and it underlies economic behavior. It describes future, uncertain events that may affect the achievement of an organization’s strategic, operational, and financial objectives (AIIA & PWC, 2006).

Actors in the market accept to take a certain level of risk in order to gain utility from their involvement in investment activities (Weyns et al., 2011). In the literature, two main approaches to measuring companies’ risk attitude can be distinguished: measures derived from the utility framework (i.e., von Neumann & Morgenstern, 1944), and measures derived from psychometrics (i.e., Miller, Kets de Vries & Toulouse, 1982). While the former approach refers to utility maximization theories found in the economic-financial literature (Schoemaker, 1982; Fishburn, 1967), the latter is focused on examining the psychological and motivational antecedents that drive market actors to accept taking some risks as a result of certain economic activities (MacCrimmon & Wehrung, 1986; Shapira, 1995).

Von Neumann and Morgenstern (1944) and Savage (1954), with their theory on the risk preferences of investors, are among the first scholars to shed light on decision making under uncertainty (Perold, 2004). Portfolio theory, by Markowitz (1952) and Roy (1952), also moves toward the consideration of risk. The question of how expected returns and expected risks are related, however, has remained, for years, unanswered by the theories of corporate finance (Perold, 2004). An initial answer is provided in the Capital Asset Pricing Model (CAPM), developed by Sharpe (1964), Lintner (1965), and Mossin (1966).

The CAPM is the first model formulated in corporate finance to establish the trade-off between the risk premium and the risk associated with investment activity (Lintner, 1965). By risk premium, we mean the premium (a higher expected rate of return on the investment) that the investor can obtain if he or she is willing to incur additional risk (Sharpe, 1964). Investors assess whether a certain risk will provide an acceptable return on investment (Riggs, 1974) in light of this trade-off. The risk/reward profile is a classic tool used to assess the acceptability of an investment, based on the evaluation of the potential profit (expected return) of an investment and the potential related losses (expected risk) (Errington, 1994; Weyns et al., 2011).

In the literature of corporate finance, there are indicators that can provide a quantitative measure of the risk/return ratio. These indicators are useful for assessing the effectiveness of an investment and for making comparisons between different instruments and solutions. Among those most used are the VaR (Value at Risk), the IR (Information Ratio) and the Sharpe Ratio. Each focuses on a particular aspect of the risk-return ratio, responding to different analysis needs (Weyns et al., 2011).

In the recent literature, the assessment of the risk/return profile of investments has been applied to sustainable investments (Siano et al., 2021), with a proposal to incorporate this indicator into corporate sustainability performance measurement systems (Searcy, 2012) or among sustainability risk management tools (Wijethilake & Lama, 2019). This raises the importance of managing sustainability-related risks and rewards especially in view of the sustainable transition (World Business Council for Sustainable Development, 2017; Wijethilake & Lama, 2019; Schulte & Hallstedt, 2018).

### Assessment of marketing investments

Marketing has always been relegated to a sporadic company expense that can be reduced or cut in case of crisis, rather than as an investment (Schultz & Gronstedt, 1997). Most recent contributions focus on the need to measure and objectify, through financial standard metrics and indicators (Stewart, 2009), the impact of marketing investments on corporate financial performance (Bolton, 2004; Hanssens & Koen, 2016; Dekimpe & Hanssens, 2018) in order to assess marketing productivity (Rust et al., 2004).

The need for greater marketing accountability and objectification of marketing investments can be seen in the words of the CMO Council (2004), which notes that marketing has always been understood more as an art than a science and has been the last of the business functions to formally develop and adopt processes and standards that can be tracked and measured quantitatively (Stewart, 2009).

In the last decade, therefore, marketing research and practice have moved towards quantitative measurements of marketing performance and investment justification (Bolton, 2004; Stewart, 2009; Hanssens & Koen, 2016). But this is done primarily by looking at the effects of it on overall company economic performance (Rust et al., 2004; Dekimpe & Hanssens, 2018). The marketing literature, in fact, indicates that traditional marketing and accounting research is interested, primarily, in static profit metrics (Hogan et al., 2002). These include return on investment, net worth, and assets. In particular, Return On Investment is the most widely used indicator, so much so that Lenskold (2002) describes ROI as the most critical measure of marketing programs for helping companies maximize profits. There are no contributions that address investment risk assessment, and the marketing literature offers insights only into systematic risk (Albers, 1998) and idiosyncratic risk (Rao & Bharadwaj, 2008) from a managerial perspective, in the context of risk management of marketing (Cavallaro, 2001).

The question of how to assess the acceptability of marketing investments (especially in ex-ante) remains open. This gap is further accentuated when discussing sustainable marketing investments.

### Sustainable marketing

In recent years, there has been a “transformative research” in marketing (Dholakia, 2009), which has led to a proliferation of marketing research towards sustainability. As such, marketing is critically reexamining its own premises and the relationship of the company not only to the natural environment, but also to society (Kilbourne, Beckmann & Thelen, 2002; Press & Arnould, 2009; Prothero, McDonagh & Dobscha, 2010).

In agreement with this transformation, Martin and Schouten (2014, p. 18) define sustainable marketing as “the process of creating, communicating, and delivering value to customers in such a way that both natural and human capital are preserved or enhanced as a whole”.

The concept of sustainable marketing (Van Dam & Apeldoorn, 1996; Gordon, Carrigan & Hastings, 2011; Kumar, Rahman & Kazmi, 2013; Lim, 2016), brings together the concepts related to social marketing (Kotler & Zaltman, 1971; Kotler & Lee, 2008) and green marketing (Henion & Kinnear, 1976; Polonsky & Rosenberger, 2001; Fisk, 1998), representing an evolution of them, but also an extension and integration of concepts related to the world of marketing and sustainability (Belz & Peattie, 2013).

According to Lunde (2018), sustainable marketing involves not just the complex exchange of value between companies and consumers, but also society and the environment (Mittelstaedt et al., 2014) so the company can meet the needs of all ecosystems around, as well as achieve its goals (Fuller, 1999; Emery, 2012). It is aimed at reducing environmental damage (Polonsky, 2011) and increasing the well-being of current stakeholders and future generations (Brundtland Commission, 1987). It promotes sustainable lifestyles, behavioral changes, and motivates consumers to adapt to sustainable products and services (Peattie 2001; Gordon et al., 2011; Rettie et al., 2012; Kemper & Ballantine, 2019), contributing to creating sustainable development and a sustainable economy (Hunt, 2011).

Some studies focus on defining how sustainable marketing has positive impacts on the sustainable economy (Chouinard, Ellison & Ridgeway, 2011; Bermejo, 2014; McDonagh & Prothero, 2014), being able to improve the economic, social and environmental performance of a company. The work of Siano et al. (2021), inspired by this consideration, reflects conceptually on the role played by sustainable marketing strategies in influencing the risk-reward profile of sustainable initiatives supported by sustainability-oriented companies. At the empirical level, however, the few studies that exist are limited to assessing the impact of sustainable marketing initiatives on consumer choices (i.e., Raju & Lonial, 2002; Lii, Wu & Ding, 2013). Moreover, there is no research in the sustainable marketing literature on the topic of sustainable marketing investments that can direct companies to the proper assessment of them.

## Method and research questions

We use a theory synthesis design in this conceptual paper (Jaakkola, 2020). A theory synthesis paper integrates multiple theories or streams of literature in order to offer a novel view of a concept. It can also characterize a previously unexplored concept, or outline a concept not yet well structured in previous literature. It is particularly useful, in fact, when research on a given topic is fragmented across different literatures or disciplines of study (Cropanzano, 2009). Researchers use theory integration to connect theoretical foundations previously considered distinct in order to reach a better understanding of a concept or phenomenon (MacInnis, 2011; Jaakkola, 2020).

We should also clarify the fine line that distinguishes a theory synthesis from a literature review tout court. For the purposes of a theory synthesis, the literature review is a key element. But it is only used as a tool, not as the ultimate goal.

Following Jaakkola (2020) suggestions, in fact, in our conceptual paper the role of the literature review is to bring out the theoretical background necessary to delineate a concept not yet addressed in the literature, namely that of sustainable marketing investments and their ex-ante assessment. We seek to advance knowledge and shed light on this unexplored concept (MacInnis, 2011), while simultaneously addressing the need, expressed in the literature, for greater marketing accountability (Hanssens & Koen, 2016; Dekimpe & Hanssens, 2018).

With this purpose, our conceptual paper attempts to answer the following research questions:

- *RQ1* What is meant by a sustainable marketing investment?
- *RQ2* How can companies assess ex-ante a sustainable marketing investment?
- *RQ3* How can companies benefit from ex-ante assessment of sustainable marketing investment?

## Ex-ante assessment of sustainable marketing investments

### Sustainable marketing investments

Investments in sustainable marketing can be a winning strategic and operational move for companies looking to transition to sustainability. The strategic importance of sustainable marketing should lead companies and research to focus more on issues related to sustainable marketing investments. In the literature, we do not yet have a definition of sustainable marketing investment. In addition, we do not have any references capable of defining the relevant parameters for evaluating marketing investment according to the changing contexts of sustainability.

Based on the definition of sustainable marketing and investment, found in the literature (and discussed in the conceptual background section), sustainable marketing investment is understood to be (RQ1):

…all those corporate initiatives aimed at the allocation of tangible and intangible resources for the implementation of projects related to strategic or operational marketing. These projects are designed to improve the environmental and social impact and the economic and financial performance of a company.

Some examples of sustainable marketing investments can include sustainable product design, the use of eco-friendly and 100% recyclable packaging (e.g., Nestlé and Barilla); solutions for delivering products to customers in order to reduce CO2 emissions, such as in the “Shipment Zero” project of Amazon; sustainable supply chain methods; the creation of sustainable points of sale, such as Heineken’s Greener Bars or Starbucks Greener Stores; and sustainable corporate communication.

Many companies, for example, focus on investments in sustainable advertising or in the creation and design of sustainable campaigns for sustainable corporate communication (i.e., traditional advertising, e-advertising, eco-labels and green certification) (Eng & Keh, 2007; Tellis, 2003).

Sustainable marketing investment qualifies as a corporate investment, so it should be evaluated and objectified not only through standard economic-financial metrics, but also through risk and profitability assessment tools common to all corporate investments.

### The risk/reward ratio for ex-ante assessment of sustainable marketing investments

The corporate finance literature emphasizes evaluating investment in terms of profitability and riskiness, that is, assessing in advance the expected risks and returns of an investment activity. Since sustainable marketing investments can be included among corporate investments we propose to use the risk/return profile for the ex-ante assessment by companies of a sustainable marketing investment (RQ2).

A sustainable marketing investment should be evaluated before implementation in order to “predictively” analyze its expected risks and rewards. Based on a comparison of these elements, it allows a decision to be made on the acceptability of the investment in terms of effectiveness and impact on a company’s reputation. This also means that one can compare various hypotheses about sustainable marketing investments and choose the most convenient one based on risk/reward/time.

In our paper we adapt the risk/reward ratio (RRr) as a tool for ex-ante assessment of sustainable marketing investments. In accordance with Riggs (1974), the use of the RRr addresses the need to have a useful indication of the acceptability of an investment. For the purpose of assessing a sustainable marketing investment, this ratio promotes the marriage of marketing and finance.

The operationalization of the risk/reward ratio implies expressing: (1) the risk of the investment in terms of the expected income loss associated with its occurrence; (2) the reward in terms of the expected net income (profit) generated by the investment. In fact, as seen, one of the key principles of the discipline of corporate finance management suggests that capital investment in markets has some degree of risk and investors should be compensated if they intend to take on that risk (Errington, 1994).

The operationalization of reward-risk leads to the following ratio:

$${\frac{\text{Expected income loss}}{\text{Expected net income}}}$$

Expected income loss is the total of all expected losses associated with the sustainable marketing investment risk. Expected net income is the difference between total estimated revenue and total estimated costs and expenses.

The implementation of the risk/reward ratio indicates whether or not a sustainable marketing investment is worthwhile. The ratio, in fact, can take on different scores and provides different outcomes relating to the acceptability of a sustainable marketing investment. Specifically, if:

- 0 < R < 1: the sustainable marketing investment is acceptable. Within this range, the expected net income is greater than the expected loss. The acceptability of the investment increases as the value of the risk-reward ratio decreases, as the value of the ratio approaches 0;
- R = 1: minimum level of acceptability of the sustainable marketing investment (perfect balance between rewards and risks);
- R > 1: the sustainable marketing investment is unacceptable. The amount of expected net income is less than the amount of expected income loss associated with the risk.

In the next section, we will discuss the rationale for evaluating the expected risks and expected rewards of a Starbucks’ sustainable marketing investment.

### Risks and rewards of Starbucks’ “Greener Stores” project

In 2018, Starbucks invests in the “Greener Stores” project, co-developed with the World Wildlife Fund (WWF), to accelerate the transformation of retail to low-impact coffee shops that achieve reductions in carbon emissions, water use and landfill waste. The “Greener Stores” project represents an investment in sustainable marketing, which will see over 10,000 Starbucks stores worldwide branded as “Greener Stores” by 2025. We question what risk and cost considerations the Starbucks company could have made in ex-ante, if it wanted to apply RRr to this specific sustainable marketing investment. In this way, we seek to illustrate how companies can benefit from the ex-ante evaluation of sustainable marketing investments (RQ3).

In first instance, Starbucks should have estimated the expected income loss by evaluating the impact of the following risks associated with the investment:

- Risks typical of the business (i.e., operational risk, financial risk, security and fraud risk, market and competition risk etc.);
- Risks related to sustainability commitment (i.e., inadequacy of product responsibility, supply chain practices, human rights and labor practices, etc.) (Anderson & Anderson, 2009; WBCSD, 2017; Wijethilake & Lama, 2019);
- Risks related to extraordinary events (i.e., wars, terrorism, pandemics, etc.), which are determined by factors that are not easily foreseeable but may impact the investment in the medium to long term if they occur.

These risk components represent the overall risk and, in relation to them, Starbucks’ expectation of loss should be quantified in the ex-ante assessment phase of the investment. In particular, as far as sustainability risks are concerned, Starbucks could have assessed the possible risks resulting from the possible non-compliance and inadequacy of the application of the declared sustainability practices in the construction and operation of its coffee shops. Starbucks could have asked itself the following question when evaluating its sustainable marketing investment: *“what is the expected loss of revenue, relative to this sustainable marketing investment, if the company fails to deliver on its sustainability promises in the future?“*. This risk is not necessarily related to fraudulent practices or deliberate greenwashing, but is inevitably linked to reputational risk. Indeed, every investment decision has an effect on the construction of corporate reputation (Hirshleifer, 1993). Unsatisfaction, disloyalty, unfavorable word of mouth, or consumer boycotts as a result of the company sending CSR (Lii et al., 2013; Luo & Bhattacharya, 2006) and sustainability signals to the target audience that do not coincide with actual practices (Vollero, Palazzo, Siano & Elving, 2016), constitute expectations of losses derived from reputational damage (Fombrun, Gardberg & Barnett, 2000; Forstmoser & Herger, 2006). It is also essential that the investment in sustainable marketing is assessed ex-ante also from the point of view of operational and strategic feasibility (i.e., the possibility of finding natural resources, managing and ensuring sustainable supply practices, etc.).

The expected income loss is also affected by cost stickiness. The phenomenon of cost stickiness (Banker & Byzalov, 2014) in relation to sustainable marketing investments deserves some comments.

In general, an investment is defined as reversible when there is an absence of sunk costs, so that a party who owns reversible capital can at any time costlessly withdraw and either consume or reinvest it outside the relationship (Crawford, 1990). Most strategic investments, however, involve some degree of stickness (Venieris, Naoum & Vlismas, 2015) and the presence of sunk costs (Chavas, 1994). Stickness indicates an “asymmetric cost behavior whereby the magnitude of cost increases in response to an increase in the activity level is greater than the magnitude of cost decreases with a decrease in the activity level” (Habib & Hasan, 2019, p. 453). When evaluating in ex-ante a sustainable marketing investment, the company will be able to evaluate and integrate sunk costs into the total expected cost and expense of the investment. In the case of the Greener Stores investment, Starbucks could have foreseen possible sunk costs linked to the investment in its coffee shops. This was in anticipation of possible changes in trends relative to the target market and, therefore, possible cessation of business or possible change of investment destination.

Among the costs, in the case of sustainable marketing investments (as for sustainability investments), moreover, CSR-related cost stickness comes into play (Habib & Hasan, 2019). A sustainable marketing investment, inevitably, communicates certain corporate CSR-related values (Lii et al., 2013) and, in ex-ante assessment, the company must provide for certain CSR-related cost stickness. During economic downturns, companies tend to cut costs, including CSR-related investments, but these costs can exhibit stickiness especially when associated with strategic CSR (Habib & Hasan, 2019). In fact, the investment time horizon of CSR investments increases the cost stickiness of CSR investments. CSR-related cost stickiness, moreover, may increase the risk of companies investing in CSR, also generating economic disadvantages relative to other less responsible organizations (López, Garcia & Rodriguez, 2007). Sunk costs, in fact, can translate into expected losses. While companies can deliberately make decisions to allocate resources to investments in sustainable marketing, it is not so easy to subsequently reduce investments in that direction, once undertaken. Especially since the signals communicated to consumers are toward sustainable behavior, this makes the company’s commitment stronger than that of other companies that do not claim to be sustainable.

The assessment of the risk linked to extraordinary events also requires some observations. The COVID-19 pandemic falls into the latter category, being an extraordinary risk factor that actually occurred and that from 2020 had a strong impact on consumer behavior and aggregation habits (Sheth, 2020) and on corporate investments (Shen et al., 2020). If Starbucks had in 2018 considered the risk item “pandemics” during the ex-ante evaluation of the investment, it could have increased the share of expected loss (for extraordinary events) and arrived at a more appropriate calculation of the RRr to establish the convenience of the investment itself.

## Discussion and implications

In this study, we provided the definition of sustainable marketing investments and how to assess ex-ante sustainable marketing investments, which may serve as a first step in advancing this field of study and, in general, theories on sustainable marketing, even from an interdisciplinary standpoint. We hope that the proposal to use RR ratio from traditional corporate finance to assess sustainable marketing investments will stimulate more interdisciplinary studies that integrate these two disciplines of study, in order to address the growing need for greater marketing accountability.

The research work proposed in this conceptual paper involves a number of reflections related to its usefulness. The discussion leads us to consider, first of all, the advantages of using RRr as a tool for ex-ante assessment of sustainable marketing investments in strategic decision-making (Donna, 2019) and in risk management strategies related to marketing activities (Cavallaro, 2001). Identifying and estimating in ex-ante the expected losses related to the risks of a sustainable marketing investment leads to assessing in ex-ante the possible future risks related to an investment, with not insignificant implications in the managerial domain.

This practice not only allows the company to protect itself from possible bad investments and the resulting economic and reputational damage (Fombrun et al., 2000; Forstmoser & Herger, 2006), but it can increase the effectiveness of strategic decision making with respect to an investment that is deemed acceptable. The ex-ante assessment of a sustainable marketing investment that is acceptable in terms of its expected risks in relation to its expected rewards can lead managers to aim to minimize the overall risk identified, through flexible strategic adaptation and the development of possible risk management strategies (Shapiro & Sheridan, 1985). The assessment of the vulnerability of the investment to the identified risk factors could, therefore, support risk management and it could be associated with the development of strategies and actions to counteract, should they occur, the possible negative effects of the risk.

In addition, the proposed use of the RRr as an ex-ante tool for evaluating sustainable marketing investments may offer support for the necessary improvement of managers’ training towards sustainable transition (Schulte & Hallstedt, 2018). In agreement with Wiek et al. (2011) and Rieckmann (2012), among the critical skills that must be imparted to company managers in view of changes towards sustainability is anticipatory thinking, in addition to systemic thinking, complexity management skills and critical thinking. In accordance with these studies, anticipatory thinking implies a change of mentality that must lead managers to “know how to think” in the long term, anticipating events and situations so as to avoid possible risks and losses. Anticipatory thinking is also closely linked to the ability to assess risks. Indeed, studies conducted on this issue show that “[…] managers are largely unfamiliar with the risks associated with sustainability and there are no supporting processes or tools to systematically work with sustainability risks” (Schulte & Hallstedt, 2018).

This unfamiliarity of managers with risk assessment reveals a serious lack of education in this area. It has negative consequences for the emergence and growth of sustainable companies. As it emerges from the literature (i.e., Aragon-Correa, Marcus, Rivera & Kenworthy, 2017; Bradfield, 2009) the need to pursue sustainable development (SD) requires specific managerial skills, which must be imparted in academic training (Rusinko; 2010; Van Kleef & Roome, 2007; Kemper, Ballantine & Hall, 2020). Managers of sustainable companies need to be trained in changing the way they have always done and seen things (Bradfield, 2009), learning to think especially in a future and predictive perspective.

## Limitations and future research

Our research has a few limitations that future studies may address. The first limitation of our conceptual work is that it is restricted to being a theoretical contribution, as it does not empirically test the validity of the tool in the ex-ante assessment of sustainable marketing investments. Future research could work in this direction with empirical studies related to the use of the risk-return ratio for the ex-ante assessment of sustainable marketing investments, making evident the applicability and advantages of this tool at the practical level. To do this, however, it is necessary to be able to count on close cooperation from companies willing to share confidential and strictly confidential data for research purposes.

A second limitation of the study is that we focus only on the risk-return ratio for the ex-ante assessment of sustainable marketing investments. We believe that this indicator can be extremely helpful in addressing the issues discussed in the study. However, we are also aware that it may not be sufficient. There is a need to be able to include additional indicators that can support evaluations of sustainable marketing investments. Future research will have to move towards the formulation or adaptation of more accurate indicators to support this evolving area of study. This will broaden the spectrum of tools that can be used in order to have increasingly adequate and reliable indications for making sustainable marketing investment decisions.
